# Synthesis and redetermination of the crystal structure of NbF_5_

**DOI:** 10.1107/S2056989023010150

**Published:** 2023-11-30

**Authors:** Martin Möbs, Florian Kraus

**Affiliations:** a Anorganische Chemie, Fluorchemie, Philipps-Universität Marburg, Hans, Meerwein-Str. 4, 35032 Marburg, Germany; University of Hyogo, Japan

**Keywords:** crystal structure, redetermination, niobium(V) fluoride, synthesis, powder X-ray diffraction

## Abstract

Single crystals of NbF_5_, niobium(V) fluoride, have been obtained by the reaction of niobium metal in a stream of dilute elemental fluorine at 473 K and subsequent sublimation. The as-obtained bulk phase compound was shown to be pure by powder X-ray diffraction at 293 K and by IR and Raman spectroscopy. A single-crystal X-ray analysis was conducted at 100 K. In comparison to the previously reported structure model [Edwards (1964 ▸). *J. Chem. Soc.* pp. 3714–3718], the lattice parameters and fractional atom coordinates were determined to much higher precision and individual, anisotropic displacement parameters were refined for all atoms.

## Abstract 2

NbF_5_ was synthesized in high purity by direct fluorination. IR and Raman spectroscopy confirms the high purity. The crystal structure was redetermined at 100 K with higher precision.

## 1. Chemical context

NbF_5_ was first synthesized by Ruff and Schiller (Ruff, 1909 ▸; Ruff & Schiller, 1911 ▸) from the reaction of Nb metal with elemental fluorine or from the reaction of NbCl_5_ with anhydrous HF. By now, several alternative ways for its synthesis have also been described in the literature (Schäfer *et al.*, 1965 ▸; O’Donnell & Peel, 1976 ▸). Niobium penta­fluoride is a colorless, hygroscopic solid that melts at 352.1 K and has a boiling point of 506.5 K (Junkins *et al.*, 1952 ▸). The vapor pressure (Junkins *et al.*, 1952 ▸; Fairbrother & Frith, 1951 ▸), the enthalpy of fusion (Junkins *et al.*, 1952 ▸), and the electrical conductivity (Fairbrother *et al.*, 1954 ▸) of liquid NbF_5_ have also been determined. Infrared and Raman spectra of the solid were measured (Preiss & Reich, 1968 ▸; Beattie *et al.*, 1969 ▸) and the structure of NbF_5_ in the (supercooled) liquid, glassy state and the gas phase have been investigated by Raman spectroscopy (Boghosian *et al.*, 2005 ▸; Papatheodorou *et al.*, 2008 ▸). In a search for a suitable laboratory synthesis of NbF_5_, we investigated several methods for its preparation. During our efforts, single crystals of several millimeters in size were obtained when hot NbF_5_ re-sublimed at colder parts of our reaction setup (see *Synthesis and crystallization*). The former crystal structure published by Edwards (1964 ▸) is of lower precision compared to structure determinations possible nowadays and displacement parameters had not been refined anisotropically.

## 2. Structural commentary

**Figure 1:**
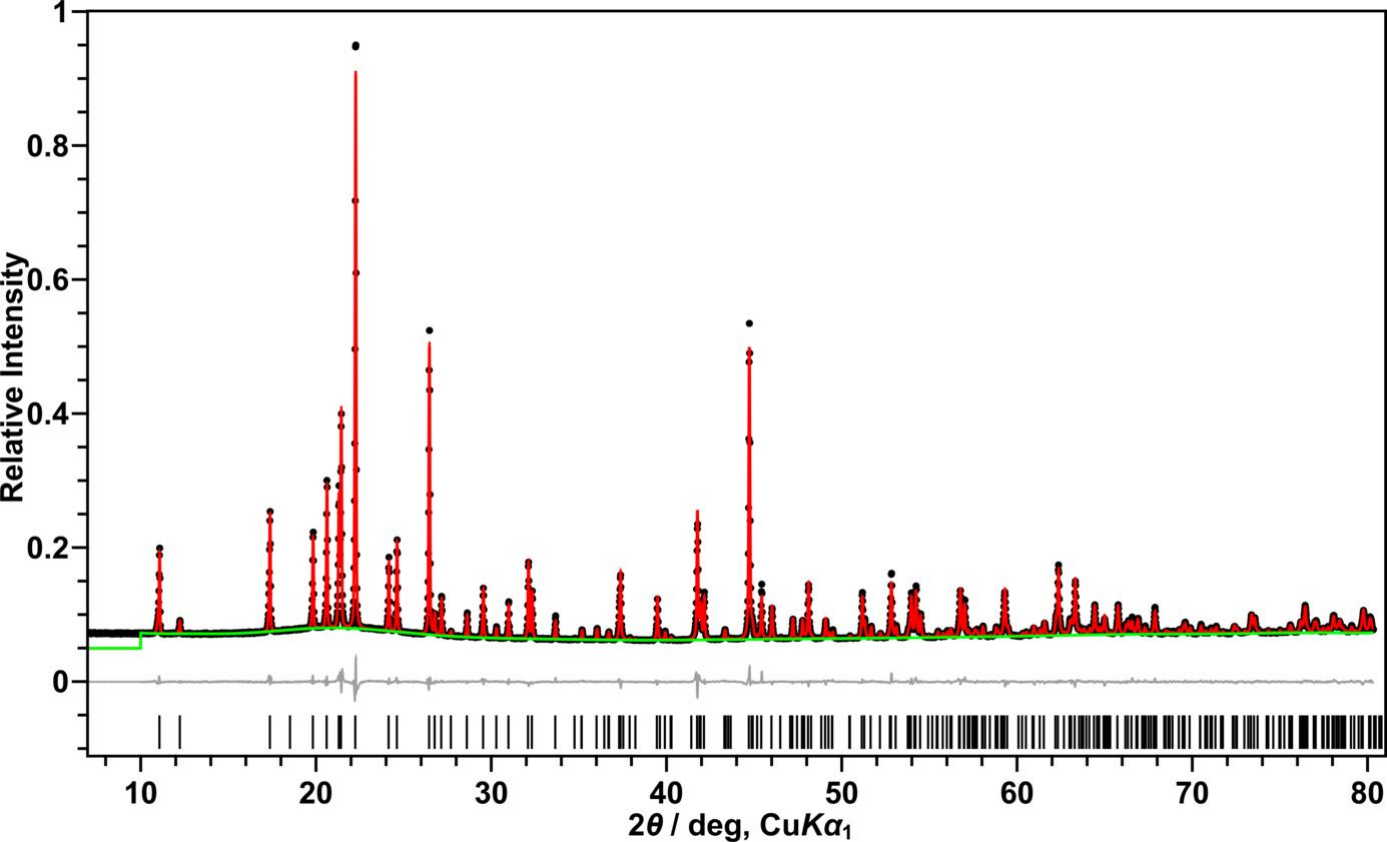
Powder X-ray diffraction pattern and Rietveld refinement of NbF_5_: measured data points (black dots), calculated diffraction pattern (red line), background (green line) and difference curve (gray). The calculated reflection positions are indicated by the vertical bars at the bottom. *R*_p_ = 3.08*, R*_wp_ = 4.25%, *R*_Bragg_ = 1.32%, *S* = 1.77.

The lattice parameters obtained from powder X-ray diffraction at 293 K [*a =* 9.62749 (19), *b* = 14.4564 (3) *c* = 5.12831 (10) Å, *β* = 95.8243 (4)°] agree with those determined by Edwards (1964 ▸) [*a =* 9.62 (1), *b* = 14.43 (2), *c* = 5.12 (1) Å, *β* = 96.1 (3)°]. Although the temperature was not explicitly stated in Edwards’ work, it can be assumed that the structure was determined at room temperature. The powder X-ray diffraction pattern is shown in Fig. 1 ▸; crystallographic details of the Rietveld refinement are given in Table 1 ▸ and the supporting information.

The single-crystal structure determination was performed at 100 K and thus resulted in smaller lattice parameters by about 1–3% compared to those determined at room temperature (see Table 1 ▸). Otherwise, there are no significant structural differences compared to the RT structure. The slight contraction of the lattice parameters is mainly due to the shortening of the distances between the Nb_4_F_20_ mol­ecules, while the intra­molecular F—Nb distances determined at 100 K differ only insignificantly from those determined at room temperature.

**Figure 2:**
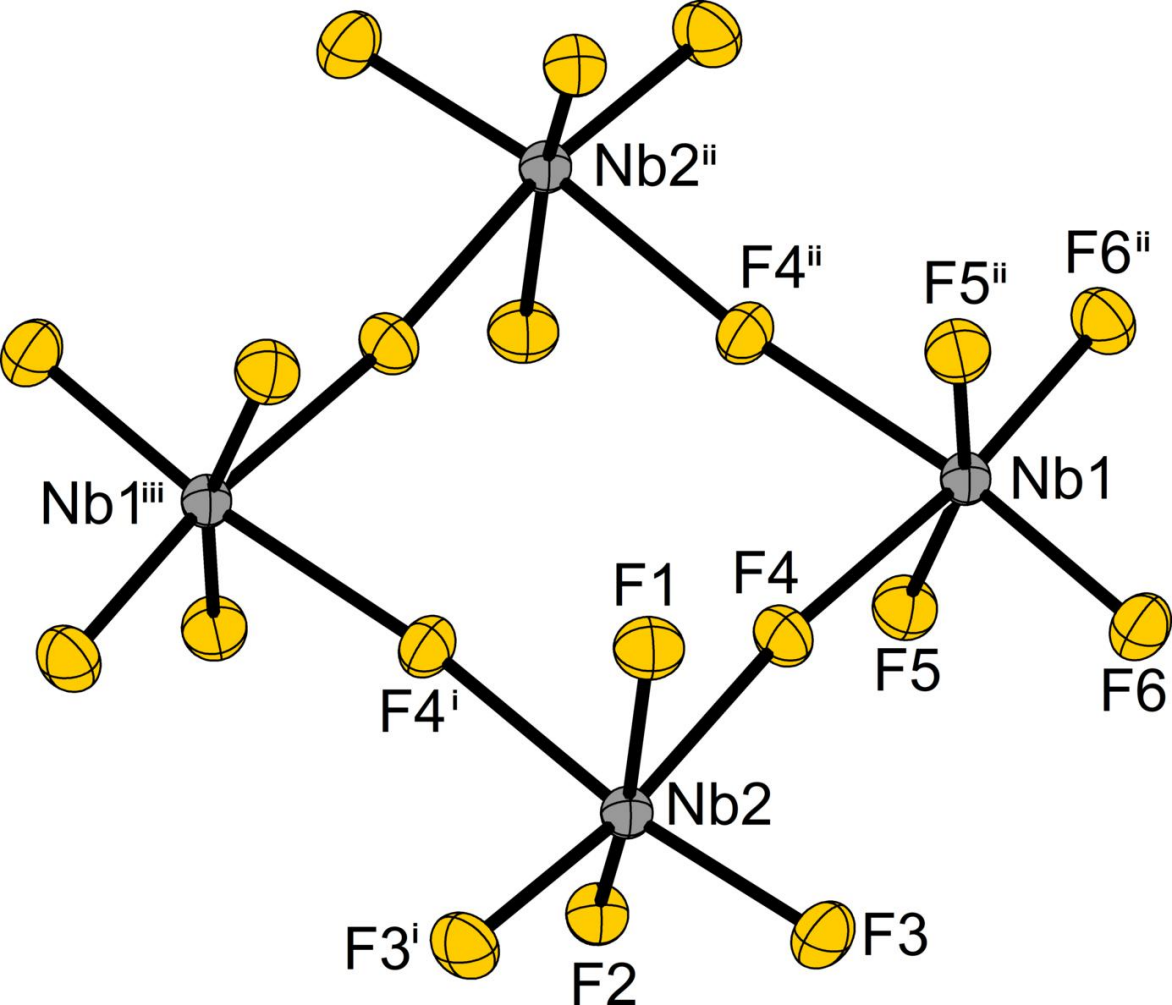
Structure of the Nb_4_F_20_ mol­ecule as it appears in the crystal structure of NbF_5_. Atom labeling in accordance with Edwards *et al.* (1962 ▸). Displacement ellipsoids are shown at the 70% probability level at 100 K. [Symmetry codes: (i) *x*, −*y*, *z*; (ii) −*x*, *y*, 1 − *z*; (iii) −*x*, −*y*, 1 − *z*.]

NbF_5_ crystallizes in the space group *C*2/*m* (No. 12, Pearson code *mC*48, Wyckoff sequence *j* ^4^ *i* ^3^ *h*) with the lattice parameters *a* = 9.4863 (12), *b* = 14.2969 (12), *c* = 4.9892 (6) Å, *β* = 97.292 (10)°, *Z* = 8 at 100 K. NbF_5_ crystallizes in the MoF_5_ structure type (Edwards *et al.*, 1962 ▸; Stene *et al.*, 2018 ▸). The structure consists of NbF_5_ units forming tetra­meric mol­ecules that can be described by the Niggli (Niggli, 1945 ▸) formula ^0^_∞_{[NbF_2/2_F_4/1_]_4_}. The structure of the Nb_4_F_20_ mol­ecule in the solid and the crystal structure of the compound are shown in Figs. 2 ▸ and 3 ▸. Two symmetry-independent niobium atoms reside on Wyckoff positions 4*h* (site symmetry 2, Nb1) and 4*i* (site symmetry *m*, Nb2) and are surrounded octa­hedron-like by six fluorine atoms. By edge-linking *via* two *cis*-positioned fluorine atoms, the NbF_6_ units form square-like mol­ecules. The atomic distance between the Nb1 atom and the μ-bridg­ing fluorine atoms F4 is 2.0669 (9) Å, while the Nb2—μ-F4 distance is 2.0685 (10). Thus, both Nb—μ-F4 bond lengths are identical within their tripled standard uncertainty. The Nb1—μ-F4—Nb2 bridge is slightly bent by 172.94 (5)°, with the bridging fluorine atoms pointing towards the ring center (Wyckoff position 2*c*, site symmetry 2/*m*) of the planar Nb_4_F_20_ rings. The distances between the Nb and the F_*trans*_ atoms, Nb1—F6 and Nb2—F3, which are opposite to the μ-bridging F atoms, are 1.8157 (11) and 1.8121 (10) Å; also overlapping within the 3σ criterion. The μ-F—Nb—F_*trans*_ angles measure 172.83 (4) and 171.95 (4)°. The terminally bound fluorine ligands in axial positions (F1, F2 and F5) show slightly longer Nb—F bonds of 1.8577 (14), 1.8378 (14), and 1.8468 (10) Å compared to those oriented equatorially (F3 and F6), showing Nb—F distances of 1.8121 (10) and 1.8157 (11) Å. This phenomenon was observed to a similar extent for the structure of MoF_5_ (Stene *et al.*, 2018 ▸) and can be attributed to the structural trans effect (Coe & Glenwright, 2000 ▸; Shustorovich *et al.*, 1975 ▸). The Nb atoms in a mol­ecule lie in a flat, nearly square plane and the crystallographic point group of the Nb_4_F_20_ mol­ecule is 2/*m* (*C*_2*h*_). The intra­molecular Nb1⋯Nb2 distance is 4.1275 (4) Å while the Nb1⋯Nb2⋯Nb1 angle measures 89.62 (1)°. The distances between diagonally opposite Nb atoms in the ring are 5.8565 (8), and 5.8179 (6) Å. Thus, the four Nb atoms of the Nb_4_F_20_ mol­ecule do not form an ideal square. It is distorted in a diamond shape, which corresponds to a compression along the twofold axis of rotation. An overview of inter­atomic distances and angles in the structure of NbF_5_ is given in Tables 2 ▸ and 3 ▸. The global crystal structure can be approximately described by a cubic close-packing of the fluorine atoms, in which 1/5th of the octa­hedral voids are occupied by Nb atoms in such a way that the Nb_4_F_20_ mol­ecules are obtained (Edwards, 1964 ▸; Müller, 2009 ▸).

In addition to X-ray powder diffraction, the bulk phase was also investigated by IR and Raman spectroscopy. The obtained spectra, which are given in the supporting information, agree with those reported in the literature (Preiss & Reich, 1968 ▸; Beattie *et al.*, 1969 ▸; Papatheodorou *et al.*, 2008 ▸), and indicate a phase pure sample.

## 3. Conclusion

NbF_5_ was synthesized from F_2_ and Nb metal and obtained as a colorless, phase-pure solid and by sublimation as single crystals. The previous structure model was significantly improved with much more precise atomic coordinates and all atoms refined anisotropically, giving much better bond lengths and angles for the Nb_4_F_20_ mol­ecules.

## 4. Synthesis and crystallization

**Figure 4:**
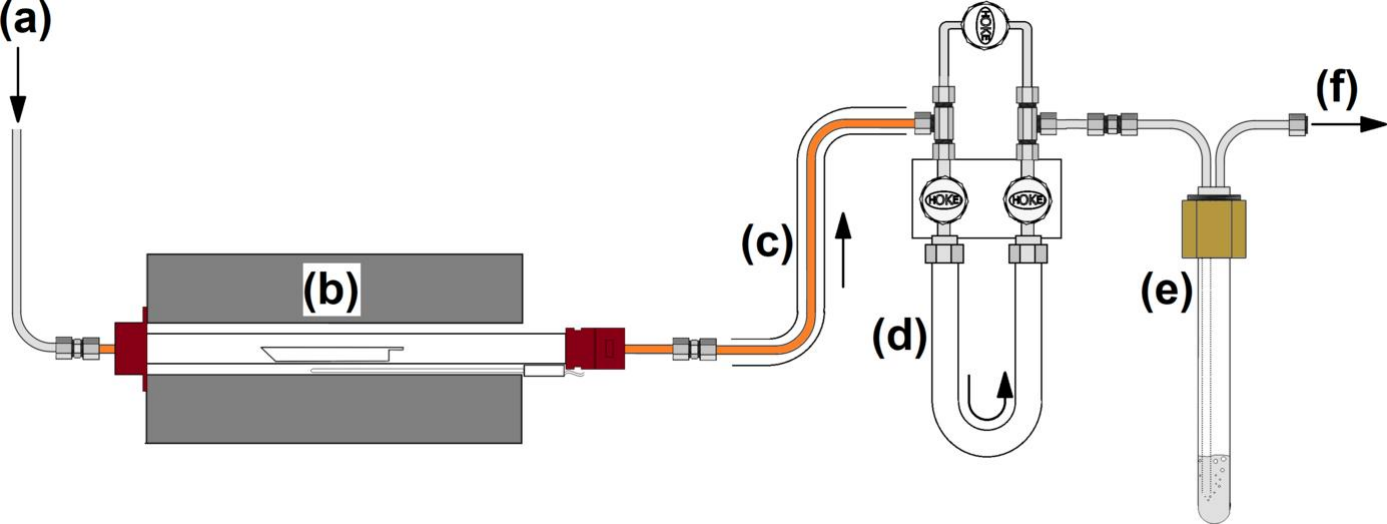
Scheme of the apparatus used for the synthesis of NbF_5_. (*a*) Connection to a metal Schlenk line for evacuation, purging with inert gas, and fluorine supply, (*b*) tube furnace, (*c*) copper pipe surrounded by a heating sleeve, (*d*) PFA U-trap for product collection equipped with Monel connectors and diaphragm valves (Hoke), (*e*) PFA gas wash bottle with steel fitting filled with perfluoro polyether, (*f*) outlet connected to the absorber.

**Figure 5:**
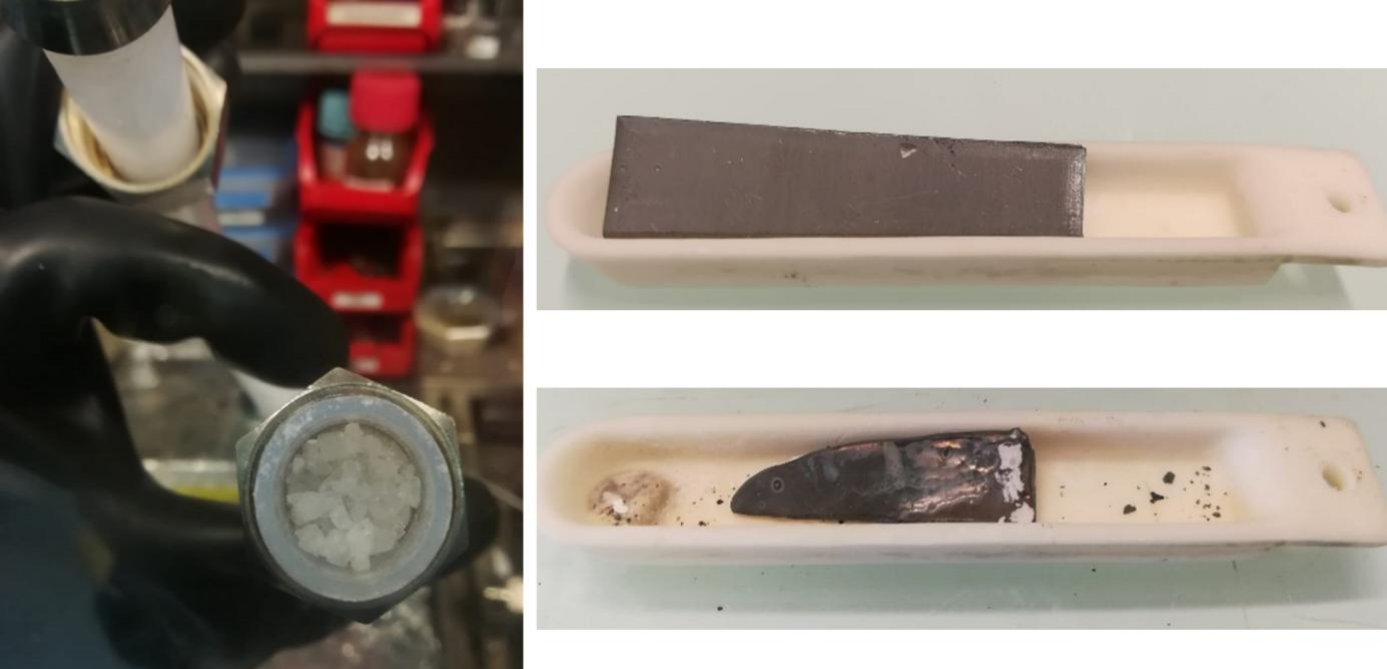
Photo of colorless crystalline NbF_5_ accumulated in the U-shaped PFA tube during the reaction (left, photo was taken inside a glove box) and corundum boat containing niobium metal: before (top right) and during the reaction (bottom right).

Niobium penta­fluoride was synthesized from the elements directly using the apparatus sketched in Fig. 4 ▸. Therein, niobium metal sheets (17.28g, 185.9mmol, TANIOBIS GmbH) were loaded in a corundum boat, which was placed inside a tube furnace. One side of the inner corundum tube of the furnace was connected to a metal Schlenk line *via* a PTFE sealed copper fitting, allowing control of the fluorine supply, as well as evacuating and purging the system with argon. The other side was connected to a U-shaped, 3/4-inch PFA tube *via* a copper pipe, followed by a PFA gas wash bottle filled with perfluoro polyether (Hostinert 216) and an absorber column filled with soda lime (Carl Roth). The copper pipe, all fittings and valves were surrounded by heating sleeves or wires and heated to 473 K to prevent resublimation of solid NbF_5_ inside. Before use, the apparatus was thoroughly baked out and passivated using diluted fluorine (F_2_/Ar, 20:80 *v*/*v*, Solvay). For the reaction a stream of diluted fluorine (F_2_/Ar, 20:80 *v*/*v*, approx. 36 mL min^−1^) was applied and the furnace temperature was set to 473 K. The first single crystals of resublimed NbF_5_ were obtained within several minutes in the U-shaped PFA tube. After 16 h the reaction was complete, giving 34.2 g (182.0 mmol, 98%) NbF_5_ as a colorless, crystalline solid (see Fig. 5 ▸).

## 5. Structure determination

*5.1 Single crystal structure determination:* A crystal of NbF_5_ was selected under pre-dried perfluorinated oil (Fomblin YR 1800) and mounted using a MiTeGen loop. Intensity data of a suitable crystal were recorded with an IPDS 2 diffractometer (Stoe & Cie). The diffractometer was operated with Mo *K*α radiation (0.71073 Å, graphite monochromator) and equipped with an image plate detector. Evaluation, integration and reduction of the diffraction data was carried out using the *X-AREA* software suite (*X-AREA* V1.90; Stoe & Cie, 2020 ▸). A numerical absorption correction was applied with the modules *X-SHAPE* and *X-RED32* of the *X-AREA* software suite. The structures were solved with dual-space methods (*SHELXT*; Sheldrick, 2015*a* ▸), and refined against *F* ^2^ (*SHELXL*) within the *ShelXle* GUI (Sheldrick, 2015*b* ▸; Hübschle *et al.*, 2011 ▸). All atoms were refined with anisotropic displacement parameters. The highest residual electron density after the final refinement was 0.80 Å distant from atom F6. Representations of the crystal structures were created with the *DIAMOND* software (Brandenburg & Putz, 2022 ▸).

*5.2 Powder X-ray diffraction:* For powder X-ray diffraction, the sample was ground using a glassy carbon mortar and filled into a quartz capillary with a diameter of 0.3 mm. The powder X-ray pattern was recorded with a StadiMP diffractometer (Stoe & Cie) in Debye-Scherrer geometry. The diffractometer was operated with Cu *K*α_1_ radiation (1.5406 Å, germanium monochromator) and equipped with a MYTHEN 1K detector.

Rietveld refinements (Rietveld, 1969 ▸) were performed using the *TOPAS-Academic* software (version 7; Coelho, 2018 ▸). The structural model derived from single-crystal X-ray diffraction was used as the starting point for the refinement. A shifted Chebyshev polynomial was used to describe the background of the powder pattern, the peak profiles were fitted with a modified Thompson–Cox–Hastings pseudo-Voigt (‘TCHZ’) function as implemented in *TOPAS*, and the zero offset was refined. To account for absorption, an intensity correction for cylindrical samples was applied as implemented in *TOPAS*. A weak preferential orientation of the crystallites was taken into account by means of a fourth-order spherical-harmonics function. The final refinement cycles converged with free refinement of all background, profile, and lattice parameters, including the coordinates of all atoms, the isotropic displacement parameters of the F atoms and anisotropic displacement parameters of the Nb atoms. Further details concerning the Rietveld refinement are given in Table 1 ▸ and in the supporting information. Crystal data, data collection and structure refinement details are summarized in Table 4. ▸

## Supplementary Material

Crystal structure: contains datablock(s) I. DOI: 10.1107/S2056989023010150/ox2001sup1.cif

Structure factors: contains datablock(s) I. DOI: 10.1107/S2056989023010150/ox2001Isup2.hkl

Rietveld powder data: contains datablock(s) I. DOI: 10.1107/S2056989023010150/ox2001Isup3.rtv

IR and Raman spectra. DOI: 10.1107/S2056989023010150/ox2001sup4.docx

CCDC references: 2309973, 2289431

Additional supporting information:  crystallographic information; 3D view; checkCIF report

## Figures and Tables

**Figure 3:**
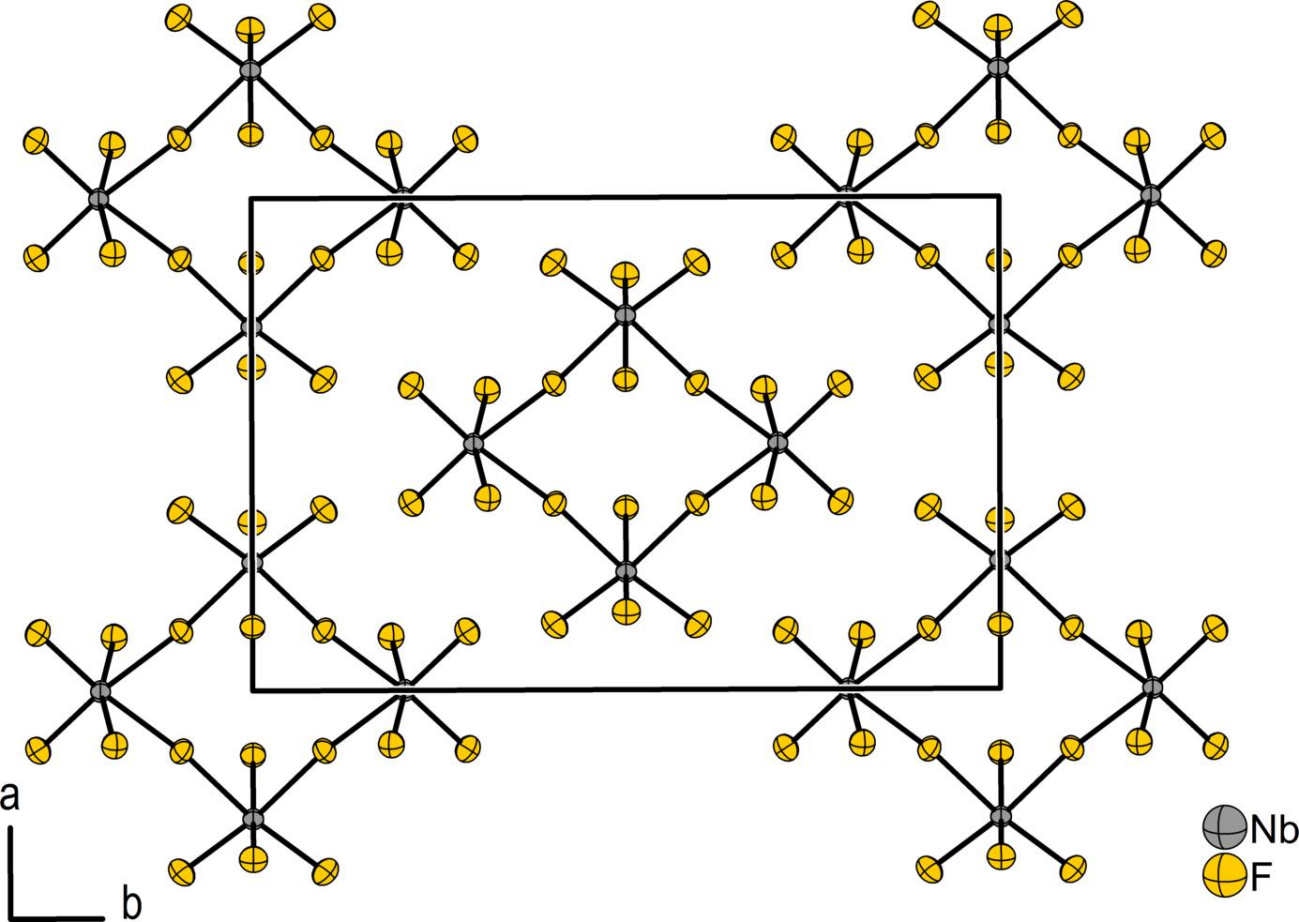
Crystal structure of NbF_5_ viewed along the *c* axis. Displacement ellipsoids are shown at 70% probability level at 100 K.

**Table 1:** Selected crystallographic details for NbF_5_ determined from single-crystal X-ray diffraction (SCXRD, middle column) and powder X-ray diffraction (PXRD, Rietveld refinement, right column)

	NbF_5_ (SCXRD)	NbF_5_ (PXRD)
Empirical formula	NbF_5_	NbF_5_
Empirical formula moiety	Nb_4_F_20_	Nb_4_F_20_
Color and appearance	colorless block	colorless powder
Size (mm^3^); capillary diameter (mm)	0.180 × 0.050 × 0.050	0.3
Mol­ecular mass (g mol^−1^)	187.91	187.91
Crystal system	monoclinic	monoclinic
Space group (No.)	*C*2/*m* (12)	*C*2/*m* (12)
Pearson symbol	*mC*48	*mC*48
*a* (Å)	9.4863 (12)	9.62749 (19)
*b* (Å)	14.2969 (12)	14.4564 (3)
*c* (Å)	4.9892 (6)	5.12831 (10)
*β* (°)	97.292 (10)	95.8243 (4)
*V* (Å^3^)	671.19 (13)	710.07 (3)
*Z*	8	8
*Z*′	2	2
ρ_calc_ (g cm^−3^)	3.719	3.515
λ (Å)	0.71073 (Mo *K*α)	1.540596 (Cu *K*α_1_)
*T* (K)	100	293
μ (mm^−1^)	3.561	27.9495
2*θ* range measured (min, max, increment)	5.182, 60.76, –	6.885, 80.340, 0.015
2*θ* range refined (min, max)	–	10.005, 80.340
*hkl*_max_	−13 ≤ *h* ≤ 13	0 ≤ *h* ≤ 8
	−18 ≤ *k* ≤ 18	0 ≤ *k* ≤ 12
	−7≤ *l* ≤7	−4≤ *l* ≤4
Absorption correction	numerical	cylindrical
*T*_max_, *T*_min_	0.7778, 0.7760	–
*R*_int_, *R*_σ_	0.0318, 0.0172	–
Completeness	0.994	–
No. of unique reflections	1048	240
No. of parameters	60	74
No. of restraints	0	0
No. of constraints	0	0
Background parameters	–	20
Profile parameters	–	12^*a*^
*R*_p_, *R*_wp_	–	0.0308, 0.0425
*R*_p_^*b*^, *R*_wp_^*b*^	–	0.0889, 0.0904
*R*_Bragg_	–	0.0132
*S* (all data)	1.024	1.77
*R(F)* [*I* ≥ 2σ(*I*), all data]	0.0143, 0.0198	–
*wR(F^2^)* [*I* ≥ 2σ(*I*), all data]	0.0315, 0.0323	–
Δρ_max_, Δρ_min_ (e Å^−3^)	0.544, −0.521	–

Notes: (*a*) refined profile parameters include spherical harmonics of order 4; (*b*) background-corrected *R*-factors.

**Table 2:** Selected inter­atomic distances (Å) for the crystal structure of NbF_5_

Nb1—F4	2.0669 (9)	Nb2—F3	1.8121 (10)
Nb1—F5	1.8468 (10)	Nb2—F4	2.0685 (10)
Nb1—F6	1.8157 (11)	Nb1—Nb2	4.1275 (4)
Nb2—F1	1.8577 (14)	Nb1—Nb1^iii^	5.8179 (6)
Nb2—F2	1.8378 (14)	Nb2—Nb2^ii^	5.8565 (8)

Symmetry codes: (i) *x*, −*y*, *z*; (ii) −*x*, *y*, 1 − *z*, (iii) −*x*, −*y*, 1 − *z*.

**Table 3:** Selected inter­atomic angles (°) for the crystal structure of NbF_5_

F6—Nb1—F6^ii^	97.52 (7)	F3—Nb2—F3^i^	98.46 (7)
F6—Nb1—F5	95.18 (5)	F3—Nb2—F2	96.78 (5)
F6^i^—Nb1—F5	95.61 (5)	F3^i^—Nb2—F2	96.78 (5)
F6—Nb1—F5^ii^	95.61 (5)	F3—Nb2—F1	94.53 (5)
F6^ii^—Nb1—F5^ii^	95.18 (5)	F3^i^—Nb2—F1	94.53 (5)
F5—Nb1—F5^ii^	163.61 (7)	F2—Nb2—F1	162.63 (6)
F6—Nb1—F4^ii^	172.83 (4)	F3—Nb2—F4	89.47 (5)
F6^ii^—Nb1—F4^ii^	89.59 (4)	F3^i^—Nb2—F4	171.95 (4)
F5—Nb1—F4^ii^	83.14 (5)	F2—Nb2—F4	83.54 (5)
F5^ii^—Nb1—F4^ii^	84.63 (4)	F1—Nb2—F4	83.43 (4)
F6—Nb1—F4	89.59 (4)	F3—Nb2—F4^i^	171.95 (4)
F6^ii^—Nb1—F4	172.83 (4)	F3^i^—Nb2—F4^i^	89.47 (5)
F5—Nb1—F4	84.62 (4)	F2—Nb2—F4^i^	83.54 (5)
F5^ii^—Nb1—F4	83.14 (5)	F1—Nb2—F4^i^	83.43 (4)
F4^ii^—Nb1—F4	83.32 (5)	F4—Nb2—F4^i^	82.57 (5)
Nb1—Nb2—Nb1^iii^	89.62 (1)	Nb1—F4—Nb2	172.94 (5)
Nb2—Nb1—Nb2^ii^	90.38 (1)		

Symmetry codes: (i) *x*, −*y*, *z*; (ii) −*x*, *y*, 1 − *z*, (iii) −*x*, −*y*, 1 − *z*.

**Table 4:** Experimental details

Crystal data	
Chemical formula	Nb_4_F_20_
*M*_r_	187.91
Crystal system, space group	Monoclinic, *C*2/*m*
Temperature (K)	100
*a*, *b*, *c* (Å)	9.4863 (12), 14.2969 (12), 4.9892 (6)
β (°)	97.292 (10)
*V* (Å^3^)	671.19 (13)
*Z*	8
Radiation type	Mo *K*α
μ (mm^−1^)	3.56
Crystal size (mm)	0.18 × 0.05 × 0.05

Data collection	
Diffractometer	Stoe IPDSII
Absorption correction	Numerical (*X-RED32* and *X-SHAPE*; Stoe & Cie, Stoe & Cie, 2020 ▸)
*T*_min_, *T*_max_	0.776, 0.778
No. of measured, independent and observed [*I* > 2σ(*I*)] reflections	5819, 1048, 903
*R*_int_	0.032
(sin θ/λ)_max_ (Å^−1^)	0.712

Refinement	
*R*[*F* ^2^ > 2σ(*F* ^2^)], *wR*(*F* ^2^), *S*	0.014, 0.032, 1.02
No. of reflections	1048
No. of parameters	60
Δρ_max_, Δρ_min_ (e Å^−3^)	0.54, −0.52

Computer programs: *X-AREA* and *X-RED32* (Stoe & Cie, 2020 ▸), *SHELXT2014/5* (Sheldrick, 2015*a* ▸), *SHELXL2018/3* (Sheldrick, 2015*b* ▸) and *DIAMOND* (Brandenburg & Putz, 2020 ▸).

## supplementary crystallographic information

### Crystal data

**Table:**

Nb_4_F_20_	*F*(000) = 688
*M**_r_* = 187.91	*D*_x_ = 3.719 Mg m^−^^3^
Monoclinic, *C*2/*m*	Mo *K*α radiation, λ = 0.71073 Å
*a* = 9.4863 (12) Å	Cell parameters from 8094 reflections
*b* = 14.2969 (12) Å	θ = 2.6–30.9°
*c* = 4.9892 (6) Å	µ = 3.56 mm^−^^1^
β = 97.292 (10)°	*T* = 100 K
*V* = 671.19 (13) Å^3^	Block, colorless
*Z* = 8	0.18 × 0.05 × 0.05 mm

### Data collection

**Table:**

Stoe IPDSII diffractometer	1048 independent reflections
Radiation source: sealed X-ray tube, 12 x 0.4 mm long-fine focus	903 reflections with *I* > 2σ(*I*)
Detector resolution: 6.67 pixels mm^-1^	*R*_int_ = 0.032
rotation method, ω scans	θ_max_ = 30.4°, θ_min_ = 2.6°
Absorption correction: numerical (*X-RED32* and *X-SHAPE*; Stoe & Cie, Stoe & Cie, 2020)	*h* = −13→13
*T*_min_ = 0.776, *T*_max_ = 0.778	*k* = −18→20
5819 measured reflections	*l* = −7→7

### Refinement

**Table:**

Refinement on *F*^2^	0 restraints
Least-squares matrix: full	Primary atom site location: dual
*R*[*F*^2^ > 2σ(*F*^2^)] = 0.014	Secondary atom site location: difference Fourier map
*wR*(*F*^2^) = 0.032	*w* = 1/[σ^2^(*F*_o_^2^) + (0.0196*P*)^2^] where *P* = (*F*_o_^2^ + 2*F*_c_^2^)/3
*S* = 1.02	(Δ/σ)_max_ = 0.001
1048 reflections	Δρ_max_ = 0.54 e Å^−^^3^
60 parameters	Δρ_min_ = −0.52 e Å^−^^3^

### Special details

**Table:**

Geometry. All esds (except the esd in the dihedral angle between two l.s. planes) are estimated using the full covariance matrix. The cell esds are taken into account individually in the estimation of esds in distances, angles and torsion angles; correlations between esds in cell parameters are only used when they are defined by crystal symmetry. An approximate (isotropic) treatment of cell esds is used for estimating esds involving l.s. planes.

### Fractional atomic coordinates and isotropic or equivalent isotropic displacement parameters (Å^2^)

**Table:**

	*x*	*y*	*z*	*U*_iso_*/*U*_eq_	
Nb1	0.000000	0.20347 (2)	0.500000	0.01085 (6)	
Nb2	0.26047 (2)	0.000000	0.24168 (4)	0.01094 (6)	
F1	0.34193 (16)	0.000000	0.6005 (3)	0.0169 (3)	
F2	0.13043 (15)	0.000000	−0.0646 (3)	0.0148 (3)	
F3	0.36790 (12)	0.09599 (7)	0.1443 (2)	0.0179 (2)	
F4	0.12213 (11)	0.09546 (6)	0.3786 (2)	0.01482 (19)	
F5	−0.10961 (12)	0.18505 (7)	0.17107 (19)	0.0163 (2)	
F6	0.11939 (13)	0.28718 (7)	0.3733 (2)	0.0171 (2)	

### Atomic displacement parameters (Å^2^)

**Table:**

	*U*^11^	*U*^22^	*U*^33^	*U*^12^	*U*^13^	*U*^23^
Nb1	0.01143 (11)	0.00957 (9)	0.01173 (9)	0.000	0.00212 (7)	0.000
Nb2	0.01106 (10)	0.01044 (9)	0.01157 (10)	0.000	0.00242 (7)	0.000
F1	0.0168 (7)	0.0195 (6)	0.0141 (6)	0.000	0.0011 (5)	0.000
F2	0.0145 (6)	0.0152 (6)	0.0145 (6)	0.000	0.0013 (5)	0.000
F3	0.0177 (5)	0.0169 (5)	0.0196 (5)	−0.0046 (4)	0.0043 (4)	0.0012 (4)
F4	0.0150 (4)	0.0130 (4)	0.0170 (4)	0.0021 (4)	0.0039 (3)	−0.0013 (4)
F5	0.0176 (5)	0.0163 (4)	0.0146 (4)	−0.0003 (4)	0.0008 (4)	−0.0004 (4)
F6	0.0175 (5)	0.0152 (4)	0.0189 (5)	−0.0026 (4)	0.0036 (4)	0.0018 (4)

### Geometric parameters (Å, º)

**Table:**

Nb1—F6	1.8157 (11)	Nb2—F3	1.8121 (10)
Nb1—F6^i^	1.8158 (10)	Nb2—F3^ii^	1.8122 (10)
Nb1—F5	1.8468 (10)	Nb2—F2	1.8378 (14)
Nb1—F5^i^	1.8468 (10)	Nb2—F1	1.8577 (14)
Nb1—F4^i^	2.0669 (9)	Nb2—F4	2.0685 (10)
Nb1—F4	2.0669 (9)	Nb2—F4^ii^	2.0685 (10)
			
F6—Nb1—F6^i^	97.52 (7)	F3—Nb2—F2	96.78 (5)
F6—Nb1—F5	95.18 (5)	F3^ii^—Nb2—F2	96.78 (5)
F6^i^—Nb1—F5	95.61 (5)	F3—Nb2—F1	94.53 (5)
F6—Nb1—F5^i^	95.61 (5)	F3^ii^—Nb2—F1	94.53 (5)
F6^i^—Nb1—F5^i^	95.18 (5)	F2—Nb2—F1	162.63 (6)
F5—Nb1—F5^i^	163.61 (7)	F3—Nb2—F4	89.47 (5)
F6—Nb1—F4^i^	172.83 (4)	F3^ii^—Nb2—F4	171.95 (4)
F6^i^—Nb1—F4^i^	89.59 (4)	F2—Nb2—F4	83.54 (5)
F5—Nb1—F4^i^	83.14 (5)	F1—Nb2—F4	83.43 (4)
F5^i^—Nb1—F4^i^	84.63 (4)	F3—Nb2—F4^ii^	171.95 (4)
F6—Nb1—F4	89.59 (4)	F3^ii^—Nb2—F4^ii^	89.47 (5)
F6^i^—Nb1—F4	172.83 (4)	F2—Nb2—F4^ii^	83.54 (5)
F5—Nb1—F4	84.62 (4)	F1—Nb2—F4^ii^	83.43 (4)
F5^i^—Nb1—F4	83.14 (5)	F4—Nb2—F4^ii^	82.57 (5)
F4^i^—Nb1—F4	83.32 (5)	Nb1—F4—Nb2	172.94 (5)
F3—Nb2—F3^ii^	98.46 (7)		

Symmetry codes: (i) −*x*, *y*, −*z*+1; (ii) *x*, −*y*, *z*.

### Selected interatomic angles (°) for the crystal structure of NbF_5_

**Table:**

F6^i^—Nb1—F5	95.61 (5)	F3^i^—Nb2—F2	96.78 (5)
F6—Nb1—F6^ii^	97.52 (7)	F3—Nb2—F3^i^	98.46 (7)
F6—Nb1—F5	95.18 (5)	F3—Nb2—F2	96.78 (5)
F6^i^—Nb1—F5	95.61 (5)	F3^i^—Nb2—F2	96.78 (5)
F6—Nb1—F5^ii^	95.61 (5)	F3—Nb2—F1	94.53 (5)
F6^ii^—Nb1—F5^ii^	95.18 (5)	F3^i^—Nb2—F1	94.53 (5)
F5—Nb1—F5^ii^	163.61 (7)	F2—Nb2—F1	162.63 (6)
F6—Nb1—F4^ii^	172.83 (4)	F3—Nb2—F4	89.47 (5)
F6^ii^—Nb1—F4^ii^	89.59 (4)	F3^i^—Nb2—F4	171.95 (4)
F5—Nb1—F4^ii^	83.14 (5)	F2—Nb2—F4	83.54 (5)
F5^ii^—Nb1—F4^ii^	84.63 (4)	F1—Nb2—F4	83.43 (4)
F6—Nb1—F4	89.59 (4)	F3—Nb2—F4^i^	171.95 (4)
F6^ii^—Nb1—F4	172.83 (4)	F3^i^—Nb2—F4^i^	89.47 (5)
F5—Nb1—F4	84.62 (4)	F2—Nb2—F4^i^	83.54 (5)
F5^ii^—Nb1—F4	83.14 (5)	F1—Nb2—F4^i^	83.43 (4)
F4^ii^—Nb1—F4	83.32 (5)	F4—Nb2—F4^i^	82.57 (5)
Nb1—Nb2—Nb1^iii^	89.62 (1)	Nb1—F4—Nb2	172.94 (5)
Nb2—Nb1—Nb2^ii^	90.38 (1)		

Symmetry codes: (i) *x*, -*y*, *z*; (ii) -*x*, *y*, 1 - *z*, (iii) -*x*, -*y*, 1 - *z*.
