# Supplementary material for: Synthesis and redetermination of the crystal structure of NbF5
Source: Acta Crystallogr E Crystallogr Commun. 2023 Nov 30;79(Pt 12):1207–11. doi: 10.1107/S2056989023010150 (PMC10833411; doi:10.1107/S2056989023010150)
Supplement: Supplementary file 4 [file e-79-01207-sup4.docx]

Supporting Information

Synthesis and Redetermination of the Crystal Structure of NbF_5_

*Martin Möbs and Florian Kraus**

M. Sc. M. Möbs, Prof. Dr. F. Kraus, Anorganische Chemie, Fluorchemie, Philipps-Universität Marburg, Hans Meerwein-Str. 4, 35032 Marburg, Germany, E-mail: [f.kraus@uni-marburg.de](mailto:f.kraus@uni-marburg.de), https://www.uni-marburg.de/de/fb15/arbeitsgruppen/anorganische_chemie/ag-kraus

# Table of contents

[Table of contents 1](#_Toc143272437)

[Vibrational Spectroscopy 2](#_Toc143272438)

[References 4](#_Toc143272439)

# Vibrational Spectroscopy

The Raman spectra were measured with a Monovista CRS+ confocal Raman microscope (Spectroscopy & Imaging GmbH) using a 532 nm solid-state laser and either a 300 grooves/mm (low-resolution mode, FWHM: <4.62 cm^−1^) or an 1800 grooves/mm (high-resolution mode, FWHM: <0.368 cm^−1^) grating. The sample was filled and sealed in a quartz capillary inside the glovebox and was then placed under the Raman microscope for data acquisition.

IR spectra were recorded on a Bruker alpha FT-IR spectrometer using the ATR Diamond module with a resolution of 4 cm^−1^. The spectrometer was located inside a glovebox (MBraun) under argon atmosphere. The spectra were processed with the OPUS software package.(OPUS V7.2, 2012)


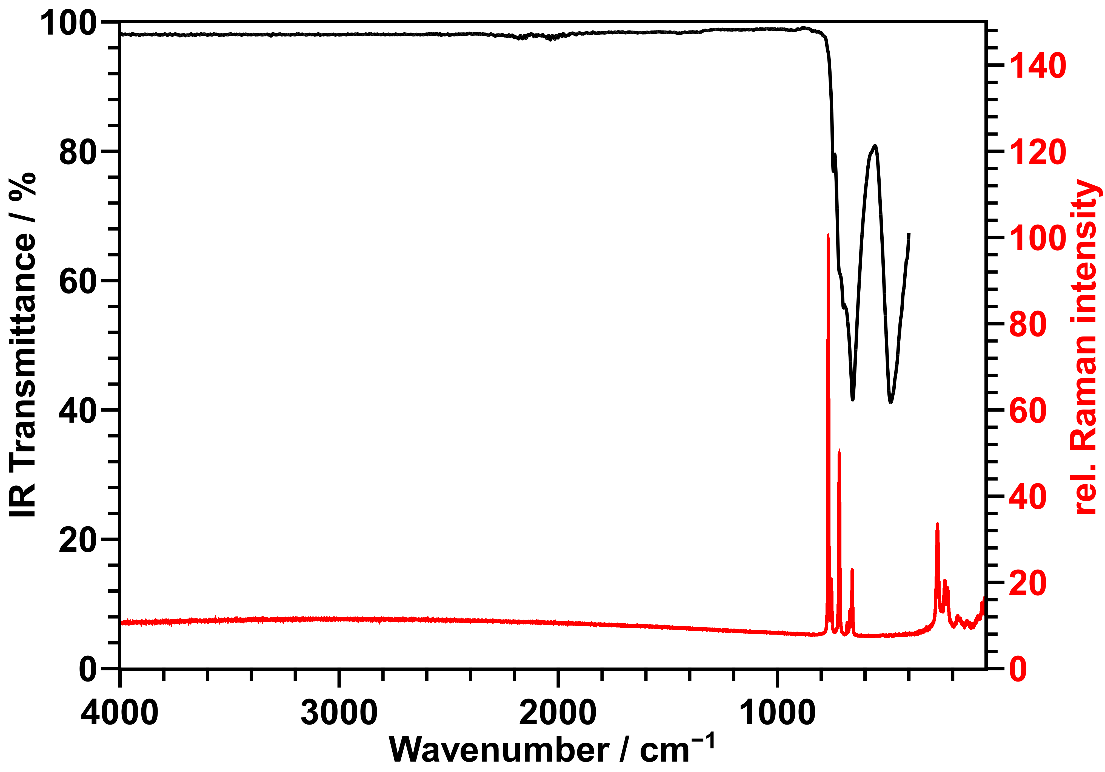


Figure S 1: Infrared (black) and Raman spectrum (red) of NbF_5_ powder.


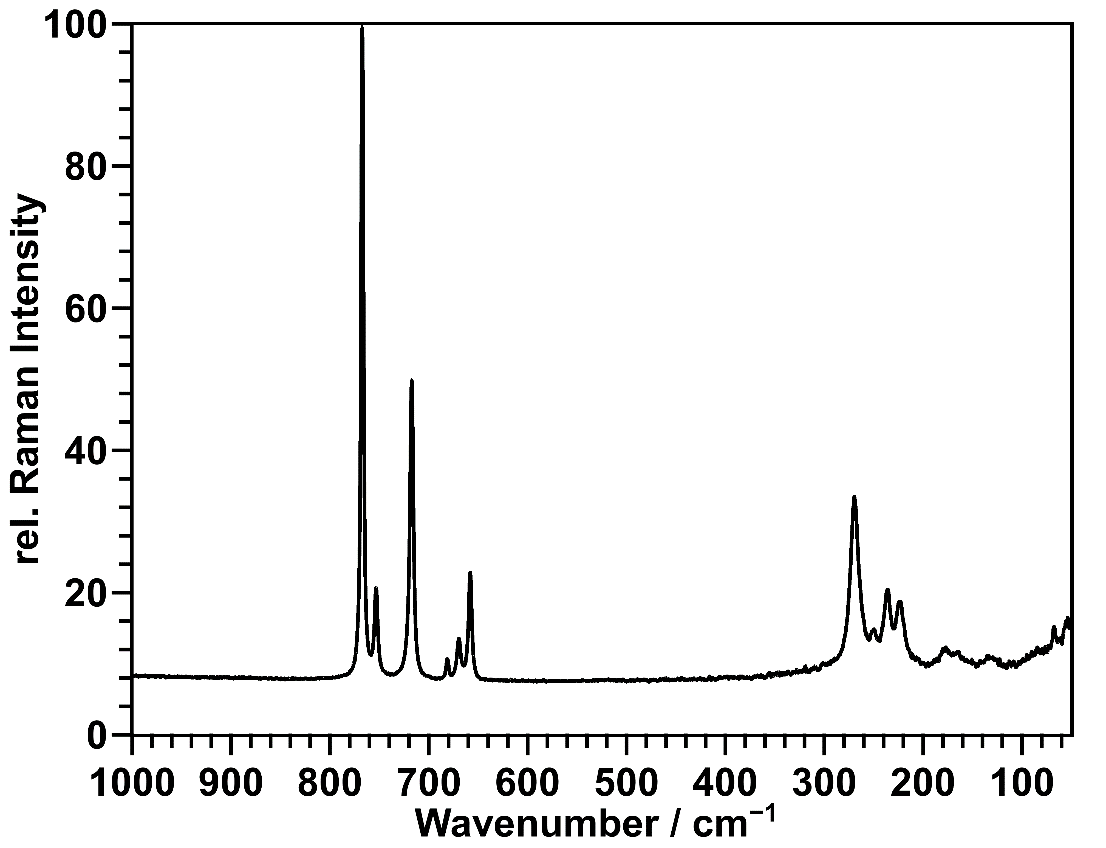


Figure S 2: Enlarged section of the Raman spectrum of NbF_5_. No further bands have been observed in the region of 1000 – 4000 cm^–1^.

Table S8. Observed vibrational frequencies for the Raman spectrum (>50 cm^–1^) and the IR spectrum (> 400 cm^–1^) of NbF_5_. Approximate band and assignment is given according to literature (Beattie *et al.*, 1969; Preiss & Reich, 1968).

| ν(observed) / cm^–1^ | | Assignment |
| --- | --- | --- |
| IR | Raman |  |
|  | 68 | lattice vibration |
|  | 133 | Nb–F deformation |
|  | 165 |  |
|  | 179 |  |
|  | 224 | Ring-deformation |
|  | 236 |  |
|  | 250 |  |
|  | 270 |  |
| 483 |  | Nb–F stretching |
| 656 | 658 |  |
|  | 669 |  |
|  | 681 |  |
| 698 | 718 |  |
| 718 | 753 |  |
| 745 | 767 |  |

# References

Beattie, I. R., Livingston, K. M. S., Ozin, G. A. & Reynolds, D. J. (1969). *J. Chem. Soc., A* 958–965.

OPUS V7.2 (2012). Ettlingen, Germany: Bruker Optik GmbH.

Preiss, H. & Reich, P. (1968). *Z. Anorg. Allg. Chem.* **362**, 19–23.
